# Ginsenoside Rg1 interferes with the progression of diabetic osteoporosis by promoting type H angiogenesis modulating vasculogenic and osteogenic coupling

**DOI:** 10.3389/fphar.2022.1010937

**Published:** 2022-11-17

**Authors:** Wenhui Chen, Xinyan Jin, Ting Wang, Rui Bai, Jun Shi, Yunxia Jiang, Simin Tan, Ruijie Wu, Shiqi Zeng, Hongxiang Zheng, Hongyang Jia, Shuanglei Li

**Affiliations:** ^1^ School of Graduate, Guangxi University of Chinese Medicine, Nanning, China; ^2^ Department of Endocrinology, The First Affiliated Hospital of Guangxi University of Chinese Medicine, Nanning, China; ^3^ Faculty of Chinese Medicine Science, Guangxi University of Chinese Medicine, Nanning, China; ^4^ School of Public Health and Management, Guangxi University of Chinese Medicine, Nanning, China

**Keywords:** type H vessels, Notch, Noggin, VEGF, diabetic osteoporosis, angiogenic-osteogenic coupling, ginsenoside Rg1

## Abstract

Ginsenoside Rg1 (Rg1) has been demonstrated to have antidiabetic and antiosteoporotic activities. The aim of this study was to investigate the protective effect of Rg1 against diabetic osteoporosis and the underlying mechanism. *In vitro*, we found that Rg1 increased the number of osteoprogenitors and alleviated high glucose (HG) induced apoptosis of osteoprogenitors by MTT assays and flow cytometry. qRT‒PCR and western blot analysis suggested that Rg1 can also promote the secretion of vascular endothelial growth factor (VEGF) by osteoprogenitors and promote the coupling of osteogenesis and angiogenesis. Rg1 can also promote the proliferation of human umbilical vein endothelial cells (HUVECs) cultured in high glucose, enhance the angiogenic ability of endothelial cells, and activate the Notch pathway to promote endothelial cells to secrete the osteogenesis-related factor Noggin to regulate osteogenesis, providing further feedback coupling of angiogenesis and osteogenesis. Therefore, we speculated that Rg1 may have similar effects on type H vessels. We used the Goto-Kakizaki (GK) rat model to perform immunofluorescence staining analysis on two markers of type H vessels, Endomucin (Emcn) and CD31, and the osteoblast-specific transcription factor Osterix, and found that Rg1 stimulates type H angiogenesis and bone formation. *In vivo* experiments also demonstrated that Rg1 promotes VEGF secretion, activates the Noggin/Notch pathway, increases the level of coupling between type H vessels and osteogenesis, and improves the bone structure of GK rats. All of these data reveal that Rg1 is a promising candidate drug for treating diabetic osteoporosis as a potentially bioactive molecule that promotes angiogenesis and osteointegration coupling.

## 1 Introduction

Diabetes mellitus (DM)-induced osteoporosis is a systemic metabolic bone disease. It causes systemic bone loss and bone microstructure damage due to chronic long-term hyperglycemia, resulting in increased bone fragility, decreased bone strength, and fracture (Parizad et al., 2019). At present, it is generally recognized that the fracture risk of type 1 diabetes mellitus (T1DM) and type 2 diabetes mellitus (T2DM) patients is significantly increased (Moayeri et al., 2017), while the proportion of osteoporosis among patients with microvascular complications of TIDM is higher (Campos Pastor et al., 2000). In the setting of microvascular disease, T1DM patients have obvious progressive bone loss, and the loss of cancellous and cortical bone occurs earlier than in T1DM patients without microvascular disease and healthy people. T2DM patients with microangiopathy show typical age-related bone loss, which is most obvious in the cortical bone (Shanbhogue et al., 2017). At the same time, high-resolution peripheral bone quantitative CT (HR-pQCT) showed that when T1DM/T2DM was combined with microvascular lesions, the bone structure of the radius was damaged, but the pathological features of T1DM were trabecular damage and a thin cortex, while the pathological features of the T2DM radius were a thin and porous cortex with an intact trabecular bone (Shanbhogue et al., 2016). Regardless of T1DM or T2DM, there is bone cortex damage in the presence of microvascular disease. The Haversian system of the Bone Cortical Reconstruction Center is the pathway of blood vessels, and microvascular disease may lead to disorders of Haversian system reconstruction, which then affects bone cortex reconstruction. Therefore, diabetes osteoporosis may be a type of microvascular disease of diabetes.

Kusumbe et al. (2014) found bone microvessels with unique morphological and functional features in the bone system, which are called type H vessels and are characterized by high expression of the endothelial markers Emcn and CD31. There is evidence that type H vessels play an important role in bone formation, bone microstructure and the number of osteoprogenitors, and type H angiogenesis can occur simultaneously with osteogenesis (Ramasamy et al., 2014). Type H vessels make the vascular environment rich in factors that favor osteogenesis and stimulate proliferation and differentiation of osteoprogenitors in the bone marrow, thereby directing bone formation (Kusumbe et al., 2014; Ramasamy et al., 2014; Xu et al., 2018). Reductions in this type of vessel are closely associated with the loss of bone mass (L. Wang et al., 2017). In conclusion, type H vessels can not only mediate the growth of bone vessels, but also maintain the level of osteoprogenitors and couple angiogenesis with osteogenesis (Kusumbe et al., 2014; Ramasamy et al., 2014; H. Xie et al., 2014). A recent study showed that T1DM in mice can lead to overall abnormalities in the intraosseous vascular system, especially type H vessels, resulting in uncoupling of angiogenesis and osteogenesis (X.-F. Hu et al., 2021). So we suspect that it may be a potential therapeutic target for high-glucose-induced bone loss.

Currently, several drugs used in clinical practice to treat osteoporosis, including bisphosphonates, estrogens, calcitonin, and denosumab, have good efficacy. However, they all achieve the therapeutic aim from the perspective of inhibiting bone resorption and fail to show beneficial effects on angiogenesis and bone formation, thus limiting their clinical application in bone loss caused by high glucose-induced microangiopathy.

Ginsenosides are the main active substances of *Panax ginseng*, *Panax quinquefolius*, and *Panax notoginseng* (Xiang et al., 2008; Kim et al., 2017). Ginsenoside Rg1 is one of the most effective components of ginsenoside. It can regulate cell proliferation, differentiation and regeneration and has anti-inflammatory, anti-apoptotic and other pharmacological activities (W. Xie et al., 2018). Previous studies have reported that ginsenoside Rg1 is able to intervene in glucose transport and disposal (C.-W. Wang et al., 2015), alter insulin secretion and bind to receptors to control blood glucose (J. Gu et al., 2013). Y. Gu et al. (2016) found that ginsenoside Rg1 could promote osteogenic differentiation of rBMSCs through BMP-2/SMAD signaling. *In vivo*, it can promote the transformation of fibrous callus into osteogenic callus and promote fracture healing in rats with tibial fracture. Another study showed that ginsenoside Rg1 is the main pro-angiogenic active component in ginseng and can increase the expression of VEGF to promote cerebral angiogenesis (Chen et al., 2019). These results suggest that ginsenoside Rg1, in addition to its anti-diabetic and anti-osteoporotic activities, also promotes angiogenesis. We therefore hypothesized that ginsenoside Rg1 may promote type H angiogenesis and interfere with angiogenic and osteogenic coupling, and reverse diabetes osteoporosis.

Increasing evidence suggests that some pathways are activated during angiogenic–osteogenic coupling, including the Notch pathway and the VEGF pathway (Ramasamy et al., 2014; Shen et al., 2021). Rg1 is closely related to these pathways and can treat a variety of diseases by regulating them (Ren et al., 2021), but studies on the treatment of diabetic osteoporosis have not been reported. Therefore, we carried out a study on the protective effect and potential mechanism of ginsenoside Rg1 on diabetic osteoporosis.

## 2 Materials and methods

### 2.1 Drugs and reagents

Ginsenoside Rg1 (purity ≥ 98%, molecular structural formula is shown in Supplementary Figure S1) was obtained from Shanghai Tubei Biological Co., Ltd. The MTT kit was obtained from Sigma (United States). Annexin V-FITC/PI kit was obtained from BestBio (Shanghai, China). PI kit and RIPA lysis buffer were obtained from Beyotime Biotechnology (Shanghai, China). The BCIP/NBT alkaline phosphatase chromogenic kit and Alizarin Red S staining kit were obtained from Solarbio (Beijing, China). SYBR Green I Master Mix and reverse transcription kit were obtained from Thermo (United States). Anti-VEGF antibody was obtained from Boster (Wuhan, China). Anti-NOG, anti-Notch1, anti-CD31 and anti-Osterix antibodies were obtained from Abcam (United Kingdom). Anti-Emcn antibody was obtained from Abbkine (Wuhan, China). Horseradish enzyme-labeled goat anti-rabbit IgG and horseradish enzyme-labeled goat anti-mouse IgG were obtained from ZSGB-Bio (Beijing, China). Anti-GAPDH antibody, fluorescein (FITC)-conjugated AffiniPure goat anti-rabbit IgG and Cy3-conjugated AffiniPure goat anti-mouse IgG were obtained from Proteintech (United States). Rabbit anti-CD73/PE conjugated antibody, rabbit anti-CD90/PE conjugated antibody, rabbit anti-CD14/FITC conjugated antibody and rabbit anti-CD19/FITC conjugated antibody were obtained from Bioss (Beijing, China).

### 2.2 Isolation, culture and identification of osteoprogenitors

Six-month-old SD rats were sacrificed, and both femurs were removed under aseptic conditions. After washing, cancellous bone was processed into 2–5 mm^2^ fragments and then repeatedly washed with PBS. Bone tissues were transferred into culture dishes, and 7 ml DMEM containing 10% fetal bovine serum, 100 U/ml penicillin, 100 mg/ml streptomycin, and 2 ml glutamine was added. Bone tissues were incubated in a 37°C incubator containing 5% CO_2_ for 4–6 days without changing the medium until a certain number of spindle-adherent cells appeared around the bone tissue under the microscope.

The first passage of osteoprogenitors was taken for identification by flow cytometry. CD73 and CD90 are markers of osteoprogenitors. At 80%–90% confluency, the osteoprogenitors were collected after treatment with trypsin. They were divided into two groups for identification. One group was treated with anti-CD73/PE antibody and anti-CD19/FITC antibody, and the other group was treated with anti-CD90/PE antibody and anti-CD14/FITC antibody and incubated in the dark at 4°C for 30 min. The control group was treated with nonspecific isotype antibody labeled with the same fluorescence. After incubation, the cells were washed twice with PBS, resuspended in 200 µl PBS, and detected by flow cytometry (BD-FACSVerse).

**FIGURE 1:**
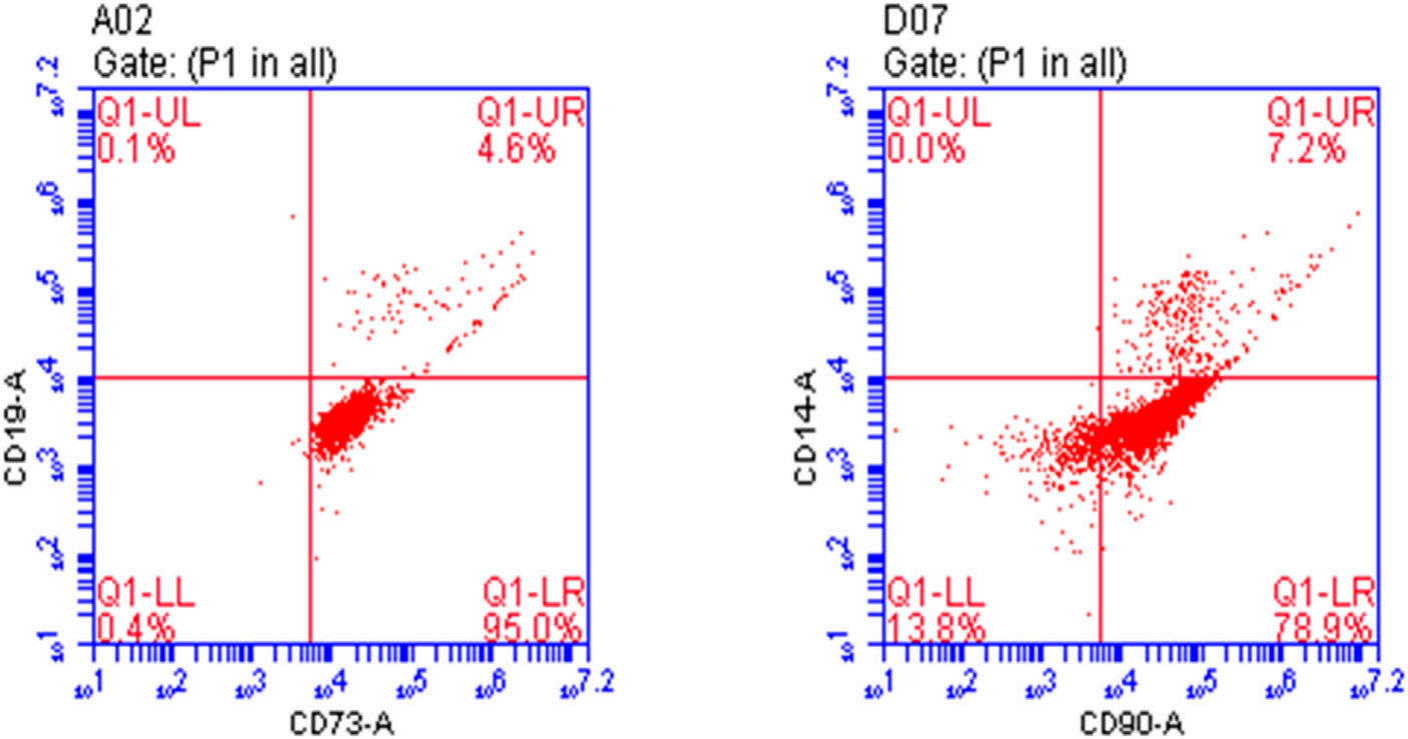
Identification of osteoprogenitors by flow cytometry.

The results showed that the positive rate of CD73 and CD90 was 99.6% and 86.1%, respectively. Osteoprogenitors lacked CD14 and CD19 expression, whereas CD19 and CD14 expression rates were 4.7% and 7.2%, indicating that the culture of osteoprogenitors was successful (Figure 1).

### 2.3 Culture of vascular endothelial cells

Human umbilical vein endothelial cells, a common cell type used for *in vitro* angiogenesis studies, were purchased from Wuxi Buhe Bio-Pharmaceutical Company. The cells were inoculated into 96-well culture plates at a density of 5 × 10^4^ cells/well for passage, and 100 μl of high-glucose DMEM was added (containing 10% fetal bovine serum, 0.1 mg/ml streptomycin and 100 U/ml penicillin). The cells were cultured at 37°C in a 5% CO_2_ incubator. At 80%–90% confluence, the cells were treated with trypsin, replated for expansion and used for downstream experiments after 4 passages.

### 2.4 Exploration of the working concentration of ginsenoside Rg1 by MTT assay

After the osteoprogenitors grew, the cell concentration was adjusted to 5 × 10^4^ cells/mL and they were inoculated into 96-well culture plates and cultured overnight at 37°C in a 5% CO_2_ incubator. Different concentrations of Rg1 were added to the cell culture wells the next day, and after 48 h of culture, 10 μl of MTT was added to each well and incubated for 3–4 h. The liquid in the wells was aspirated, 200 μl of DMSO (Invitrogen) was added, and the OD value was measured using a microplate reader (Thermo, MK3) at a wavelength of 492 nm. Using CurveExpert software, the working concentration of Rg1 was calculated to be 164.8 μM (Supplementary Table S1), and this concentration was used for the downstream experiments.

### 2.5 Proliferation of osteoprogenitors was measured by MTT assay

After the osteoprogenitors grew, the cell concentration was adjusted to 5 × 10^4^ cells/mL and they were inoculated into 96-well culture plates and cultured overnight at 37°C in a 5% CO_2_ incubator. Osteoprogenitors were divided into four groups: the CON group (with 5.5 mmol/L glucose), HG group (with 32.8 mmol/L glucose), Rg1 group (with Rg1 concentration of 164.8 uM) and HG + Rg1 group (with 32.8 mmol/L glucose + Rg1 concentration of 164.8 uM). The culture conditions except for the culture medium were kept consistent across the groups. After incubation for 24 h, 48 h and 72 h, 10 μl MTT was added to the wells of each group and then incubated for 3–4 h. OD value was measured with a microplate reader at a wavelength of 492 nm after shaking the liquid for 10 min. The measured OD values were used for the analysis of cell viability.

### 2.6 Proliferation of HUVECs were measured by MTT assay

After the HUVECs grew, the cell concentration was adjusted to 5 × 10^4^ cells/mL and they were inoculated into 96-well culture plates and cultured overnight at 37°C in a 5% CO_2_ incubator. The HUVECs were divided into three groups: the CON group (with 5.5 mmol/L glucose), HG group (with 32.8 mmol/L glucose), and HG + Rg1 group (with 32.8 mmol/L glucose + Rg1 concentration of 164.8 uM). The culture conditions except for the culture medium were kept consistent across the groups. After incubation for 24 h, 48 h and 72 h, 10 μl MTT was added to the wells of each group and then incubated for 3–4 h. OD value was measured with a microplate reader at a wavelength of 492 nm after shaking the liquid for 10 min. The measured OD values were used for the analysis of cell viability.

### 2.7 Flow cytometry detection of apoptosis of osteoprogenitors and HUVECs

Cells in each group were treated with trypsin without EDTA, and centrifuged at 1,500 rpm at room temperature for 5 min to collect cells. The cells were resuspended with PBS, then washed, and then 300 µl of 1× Binding Buffer suspension cells were added. Add 5 µl Annexin V-FITC, mix well, incubate in dark at room temperature for 15 min, and then stain with 10 µl PI. Finally, flow cytometry was used for detection and FlowJo 7.6 software was used for analysis.

### 2.8 Alkaline phosphatase (ALP) and alizarin red s (ARS) staining

ALP and ARS staining of the osteoprogenitors was used to assess the effect of ginsenoside Rg1 on osteogenic differentiation. Briefly, osteoprogenitors were inoculated in 24-well plates after adjusting the cell concentration to 1 × 10^5^ cells/ml. The osteoprogenitors were divided into three groups according to different treatment methods: control group (with 1,640 medium (Hyclone)), inducer group (with osteogenic induction medium) and ginsenoside Rg1 + inducer group (with osteogenic induction medium + Rg1 concentration of 164.8 uM). Osteogenic induction medium contained 10% FBS, 2% penicillin‒streptomycin, 0.01 µmol/L dexamethasone, 10 mmol/L β-glycerophosphate sodium, and 50 mg/L ascorbic acid. Proliferation of osteoprogenitors was observed daily under a microscope. ALP staining and activity assays were performed with a BCIP/NBT kit at 7 days after osteogenic induction, and mineral deposition was evaluated by Alizarin Red S staining at Day 14 of differentiation. Finally, ImageJ software was used for quantitative analysis.

### 2.9 Immunofluorescence staining

Osteoprogenitor samples from the above three groups were fixed in 4% paraformaldehyde and permeabilized with 0.1% Triton for 15 min. After blocking with 5% FBS for 1 h at room temperature, the cells were incubated overnight at 4°C with anti-Osterix antibody (1:1,000). Subsequently, cells were washed three times with PBS and incubated for 1 h with FITC-conjugated Affinipure Goat Anti-Rabbit IgG (1:200). Incubate with Hoechst (Beyotime Biotechnology, Shanghai, China) for 15 min at room temperature in the dark. Finally, fluorescence signals were collected using a fluorescence microscope (OLYMPUS, IX71, Japan).

### 2.10 Quantitative real-time polymerase chain reaction analysis

Total RNA of bone tissue or cells was extracted with TRIzol reagent (Invitrogen). cDNA was obtained from total RNA using a Reverse Transcription Kit. Next, qRT‒PCR was performed using SYBR Green qPCR Master Mix. Relative gene expression was calculated using the 2^−△△Ct^ method, and GAPDH was used as a reference for normalization. The real-time PCR primer sequences are shown in Table 1.

**TABLE 1:** Primer sequences used in the qRT-PCR assays.

Primer	Sequence (5′-3′)
VEGF (RAT)-RT-F	GAG​TAT​ATC​TTC​AAG​CCG​TCC​TGT​GTG
VEGF (RAT)-RT-R	GTT​CTA​TCT​TTC​TTT​GGT​CTG​CAT​TCA
GAPDH (RAT)-RT-F	ACG​ACC​CCT​TCA​TTG​ACC​TCA​ACT​ACA
GAPDH (RAT)-RT-R	GAC​ATA​CTC​AGC​ACC​AGC​ATC​ACC​CCA
Notch1 (RAT)-RT-F	ACA​GTG​CCG​AGT​GTG​AGT​GGG​ATG​G
Notch1 (RAT)-RT-R	CAG​GAA​GTG​GAA​GGA​GTT​GTT​GCG​T
NOG (RAT)-RT-F	CCA​GCA​CTA​TCT​ACA​CAT​CCG​CCC​A
NOG (RAT)-RT-R	GCG​TCT​CGT​TCA​GAT​CCT​TCT​CCT​T
GAPDH (human)-RT-F	ATG​GGG​AAG​GTG​AAG​GTC​GGA​GT
GAPDH (human)-RT-R	TAG​TTG​AGG​TCA​ATG​AAG​GGG​TC
Notch1 (human)-RT-F	GCA​GCC​TCA​ACA​TCC​CCT​ACA​AGA
Notch1 (human)-RT-R	CCC​ACG​AAG​AAC​AGA​AGC​ACA​AAG
NOG (human)-RT-F	CTT​TTG​GCC​GCG​CTA​CGT​GAA​G
NOG (human)-RT-R	TCG​GAA​ATG​ATG​GGG​TAC​TGG​A

### 2.11 Western blot analysis

Total proteins were obtained with RIPA lysis buffer (Beyotime, Shanghai, China) supplemented with phenylmethanesulfonyl fluoride. After centrifugation at 12,000 × g for 10 min, the supernatant was extracted. Proteins were resolved by 10% SDS‒PAGE and transferred to PVDF membranes (Millipore, United States) by electroblotting. Blocking was performed with 5% skimmed milk for 2 h at 37°C followed by incubation with anti-NOG, anti-Notch1, anti-VEGF (1:1000) and anti-GAPDH (1:5000) antibodies overnight at 4°C. After washing three times with TBST solution, the blots were incubated with the secondary antibodies (goat anti-mouse IgG and goat anti-rabbit IgG, 1:5000) conjugated to horseradish peroxidase for 1 h at 37°C. An appropriate amount of ECL luminescence solution (Beijing Dingguo) was added to the membrane, and images were taken using an integrated chemiluminescence instrument (ChemiScope 5300 Pro).

## 3 Animals and treatments

### 3.1 Animals and experimental design

GK rats are a polygenic non obese diabetic rat model developed by selection from Wistar rats. It is characterized by impaired glucose-stimulated insulin secretion, fasting hyperglycemia and insulin resistance, which are very similar to the development of type 2 diabetes in humans. Moreover, it was found that the bone mineral density (BMD) of the femur and fifth lumbar vertebra in 6-month-old GK rats were lower than that in normal control Wistar rats, and the bone formation indicator osteocalcin (OCN) was significantly reduced, and the bone absorption indicator tartrate resistant acid phosphatase (TRAP) activity was significantly increased, suggesting that GK rats have osteopenia and an increased risk of fracture (Zhang et al., 2009). GK rats provide a good animal model for studying the pathogenesis and preventive measures of diabetic osteoporosis.

A total of 20 female 4-month-old GK rats purchased from Covance Laboratory Animal Co., Ltd. were used in this study. They were divided into two groups by random number table: the GK rat model group and the GK rat + Rg1 group, with 10 rats in each group. Another 10 Wistar rats of the same age were used as the blank control group. A standard laboratory diet was fed to all rats with *ad libitum* access to feed and water. The GK rats + Rg1 group were intragastrically administered Rg110 mg/kg/d, and the blank control group and the GK rats group were intragastrically administered an equal volume of normal saline. Micro-computed tomography (micro-CT) examination was performed before Rg1 treatment in Wistar group and GK group for reference. After 12 weeks of treatment, all three groups of rats underwent micro-CT examination, bone tissue morphometric analysis, qRT‒PCR analysis (same method as Section 2.10), and western blot analysis (same method as Section 2.11). All experimental procedures were approved by the Animal Ethics and Experimental Safety Committee of Guangxi University of Traditional Chinese Medicine.

### 3.2 Micro-CT analysis

The dissected tibiae were soaked in tissue fixative and then analyzed with a micro-CT Skyscan 1176 (Skyscan, Aartselaar, Belgium). The scanner was set to a voltage of 50 kV, a current of 800 μA, and a resolution of 12 μm per pixel. Three-dimensional image reconstruction was performed with N-Recon software, and three-dimensional analysis was performed with CT-AN software to measure bone volume/total volume (BV/TV), trabecular thickness (Tb. Th), trabecular number (Tb. N), trabecular separation (Tb. Sp), structure model index (SMI) and BMD in each group.

### 3.3 Histological analysis

For histological analysis, after sacrificing the rats, the proximal metaphysis of the tibia of the right hind limb (approximately 1/3 of the total length) was taken, and the tibial samples were fixed in 4% paraformaldehyde solution for 24 h. They were decalcified in 7% EDTA for 2 weeks, dehydrated through graded ethanol of an increasing concentration, and then embedded in paraffin. The samples were cut into 5-μm-thick sections, deparaffinized, and rehydrated. Hematoxylin staining (Shanghai Zhanyun Chemical Co., Ltd.) was performed for 5 min followed by washing with distilled water, sections were then stained with eosin (Shanghai Zhanyun Chemical Co., Ltd.) for 2–8 s, dehydrated with absolute ethanol, added with neutral gum, and sealed. Finally, it was observed under a microscope.

### 3.4 Immunofluorescence staining

Tibial specimens were fixed in 10% formaldehyde, washed in water, then dehydrated in ethanol, embedded in paraffin, and samples were sectioned, deparaffinized in xylene, and rehydrated. Tibial specimens were incubated in 3% H_2_O_2_/PBS for 10 min to quench endogenous peroxidase activity. After antigen retrieval in trisodium citrate buffer at 95°C for 5 min and blocking with 5% BSA for 15 min, the sections were incubated with anti-CD31 antibody, anti-Emcn antibody (1:200) and anti-Osterix antibody (1:1000) at 4°C overnight. After washing with PBS, the corresponding rabbit secondary antibody or mouse secondary antibody (1:200) was added and incubated for 1 h. Finally, the slides were mounted after staining with Hoechst and observed under a microscope.

## 4 Statistical analysis

All data are presented as the mean ± standard deviation (SD). Data were analyzed using GraphPad Prism 9.0 software (GraphPad Software, United States). Two-tailed Student’s t-test was used for comparison between two groups, and Tukey’s multiple comparison test was performed after one-way analysis of variance (ANOVA) for multiple groups. The differences were judged to be statistically significant when *p* < 0.05.

## 5 Results

### 5.1 Ginsenoside Rg1 promotes osteogenic differentiation of osteoprogenitors

**FIGURE 2:**
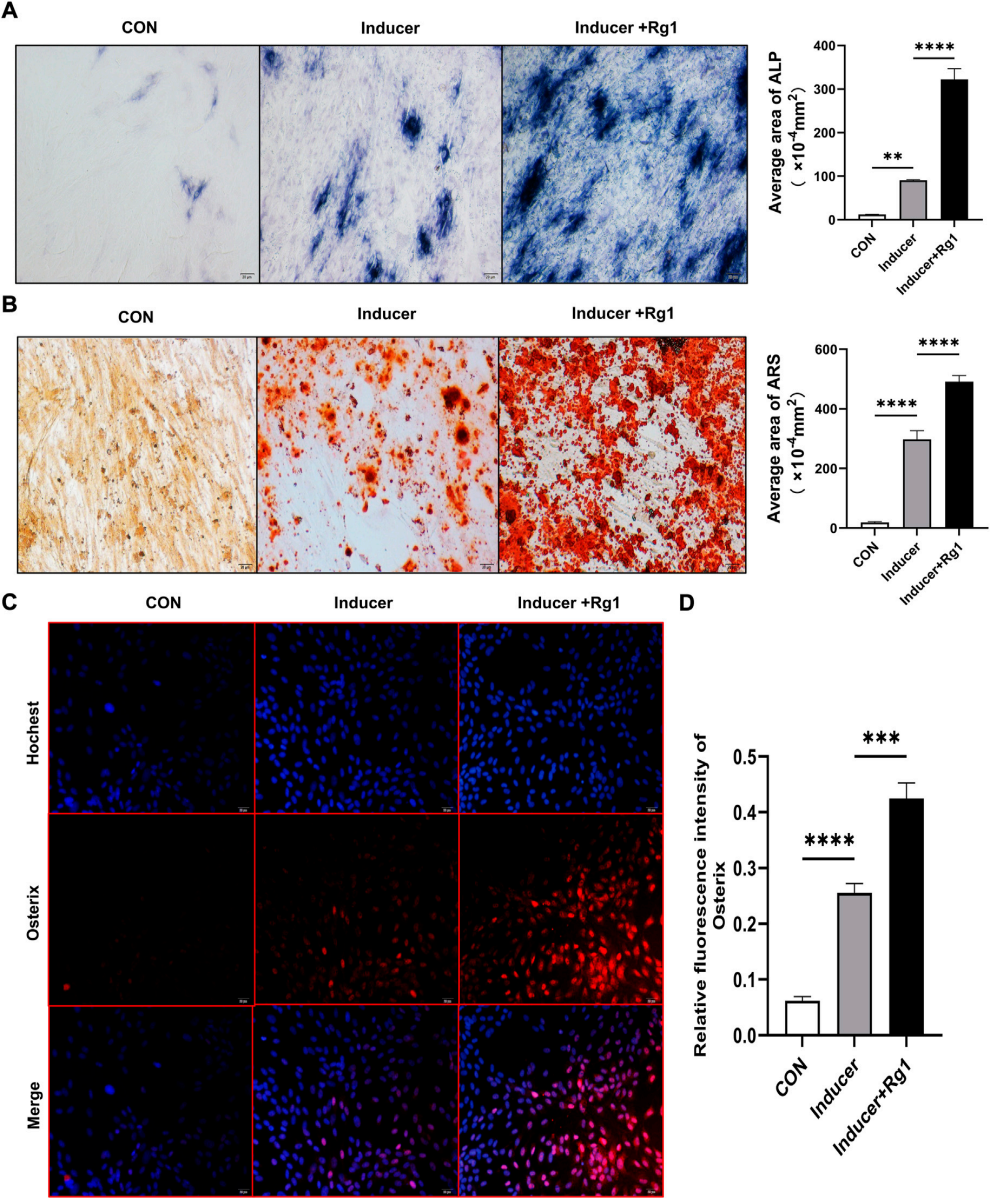
Ginsenoside Rg1 can promote osteogenic differentiation of osteoprogenitors. **(A)** ALP staining and quantitative analysis in CON group, Inducer group and Inducer + Rg1 group. **(B)** Alizarin red staining and quantitative analysis in CON group, Inducer group and Inducer + Rg1 group. **(C)** Immunofluorescence staining of Osterix in the CON group, Inducer group and Inducer + Rg1 group. **(D)** Quantification of the mean relative levels of Osterix in osteoprogenitors in each group. Scale bar: 20 µm. Data are presented as the mean ± SD. *p < 0.05, **p < 0.01, ***p < 0.001, ****p < 0.0001, NS: not significant. The above experiments were independently performed three times.

To evaluate the role of Rg1 in bone formation *in vitro*, we isolated and cultured osteoprogenitors and carried out osteogenic differentiation experiments. The results of the ALP staining (Figure 2A) showed that ALP in the control group without osteogenic inducer basically did not stain, and there was no significant osteogenic differentiation. In the Inducer group, the ALP staining was intense, and osteogenic potential was enhanced. ALP staining was further enhanced in cells cotreated with Rg1 and inducer. ALP-positive cells were significantly increased, and osteogenic induction showed the most significant effect. ARS staining showed similar results (Figure 2B), with significant staining and enhanced osteogenic potential in the Inducer group compared with the control group. Compared with the Inducer group, ARS staining was more obvious in the Inducer + Rg1 group, indicating that the number of calcium nodules was increased and the osteogenic potential was significantly enhanced.

Osterix is well-known to be an osteoblast-specific transcription factor that is mostly densely distributed around type H vessels and plays an important role in osteoblast differentiation, maturation and activity (Ramazzotti et al., 2019). The Osterix gene is a signature gene for osteoblast lineage formation. We used immunofluorescence staining to detect its expression level to further evaluate the role of Rg1 in promoting bone differentiation. The results showed (Figures 2C,D) that Osterix expression levels were low in the control group and increased after addition of the inducer. Compared with the Inducer group, the Inducer + Rg1 group had significantly increased Osterix expression levels and promoted osteogenic differentiation of cells to a greater extent.

### 5.2 Ginsenoside Rg1 can promote (under high glucose exposure) osteoprogenitor proliferation and inhibit osteoprogenitor apoptosis

**FIGURE 3:**
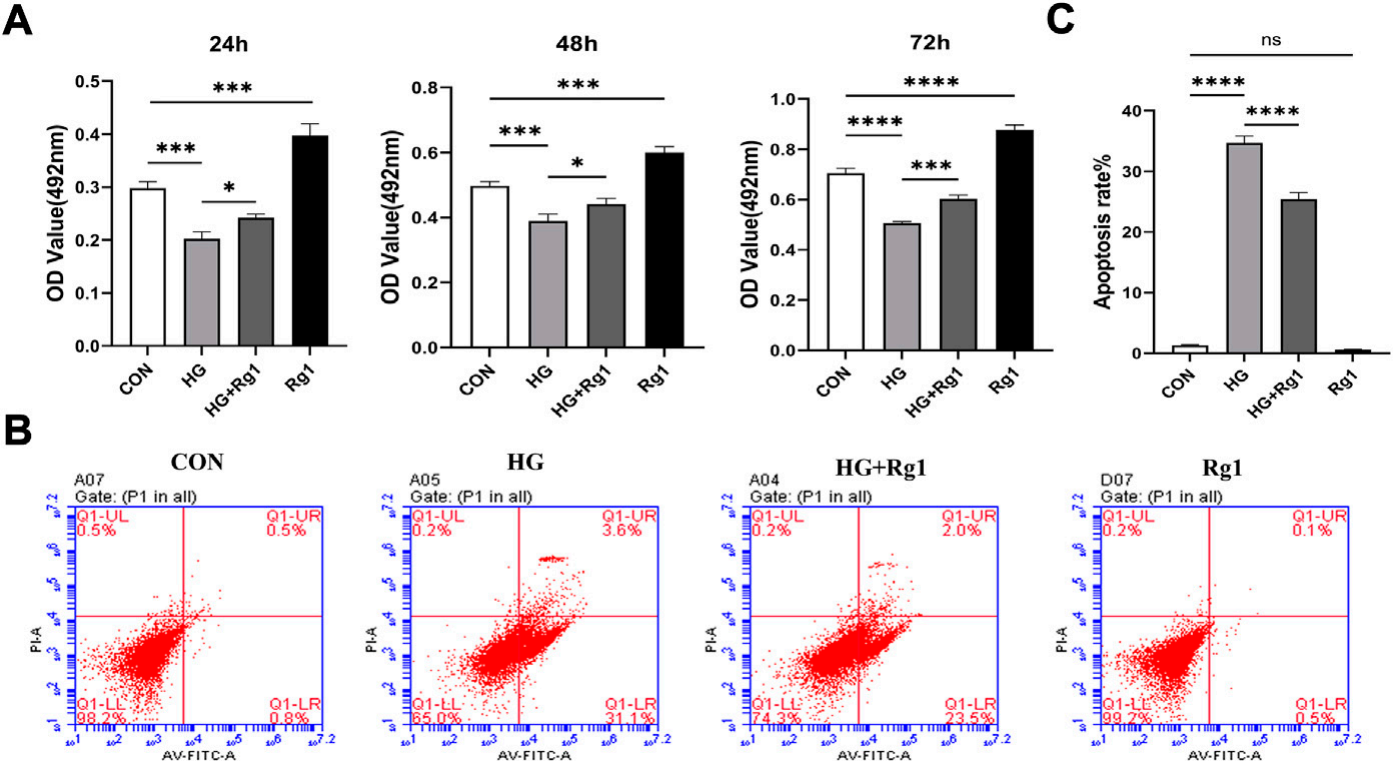
Ginsenoside Rg1 can promote (under high glucose exposure) osteoprogenitor proliferation and inhibit apoptosis. **(A)** MTT assays were used to assess the proliferation of osteoprogenitors by OD values in each group at 24 h, 48 h, and 72 h. **(B)** Flow cytometry was used to detect the apoptosis of osteoprogenitors in each group at 48 h. **(C)** The apoptosis rate of the osteoprogenitors in each group at 48 h. Data are presented as the mean ± SD. *p < 0.05, **p < 0.01, ***p < 0.001, ****p < 0.0001, NS: not significant. The above experiments were independently performed three times.

Osterix ^+^ osteoprogenitors give rise to osteoblasts and osteocytes (Kusumbe et al., 2014), and their recruitment plays an important role in bone tissue regeneration and remodeling. Lower numbers of osteoprogenitors in the bone marrow stroma may impede osteoblast generation and bone formation and compromise bone mass and healing (Weinberg et al., 2014). To investigate the effect of Rg1 on the proliferation and apoptosis of osteoprogenitors, we first detected the proliferation of osteoprogenitors treated with Rg1 for 24 h, 48 h, and 72 h by MTT assays. The results (Figure 3A) showed that the proliferation of osteoprogenitors was similar in the three time periods, and the addition of Rg1 significantly promoted cell proliferation under normal culture conditions. Compared with the control group, cell proliferation was significantly inhibited in high glucose culture, but when Rg1 was added to high glucose culture, the inhibition of cell proliferation significantly improved. The effect was most apparent at 48 h and the cells reached confluence at 72 h, so 48 h was selected for the subsequent experiments. Previous experiments have shown that the number of osteoprogenitors is significantly decreased in aged and ovariectomized mice (Kusumbe et al., 2014; L. Wang et al., 2017) and our study demonstrated that a similar situation also occurs during bone formation under high glucose exposure. As shown by flow cytometry (Figures 3B,C), compared with the control group, the apoptosis rate of osteoprogenitors was significantly increased under high glucose culture and significantly decreased when Rg1 was added under high glucose culture.

### 5.3 Ginsenoside Rg1 promotes the secretion of vascular endothelial growth factor from high glucose-exposed osteoprogenitors and regulates angiogenesis

**FIGURE 4:**
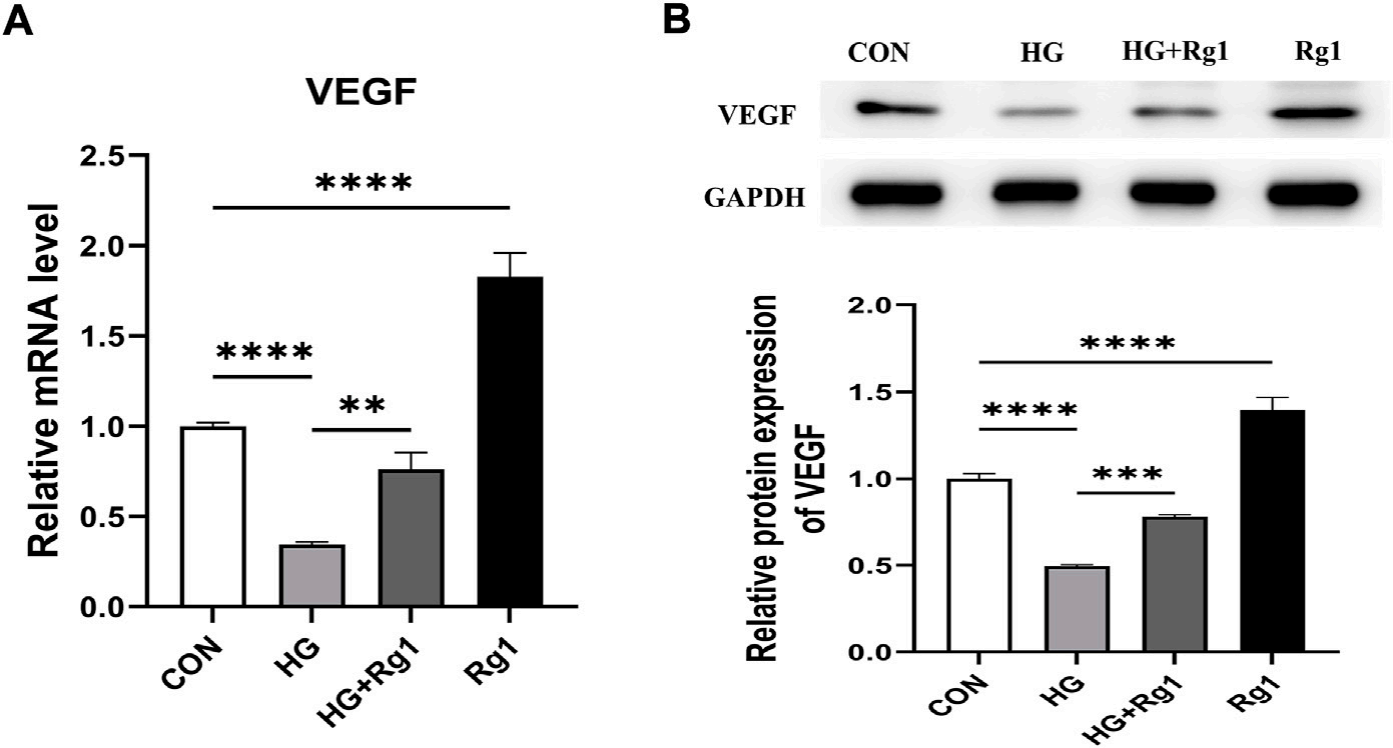
Ginsenoside Rg1 promotes VEGF secretion from osteoprogenitors. **(A)** qRT‒PCR was used to detect the expression level of VEGF mRNA in osteoprogenitors in each group. **(B)** Western blotting was used to detect the expression level of VEGF protein in osteoprogenitors in each group. The housekeeping gene GAPDH served as an internal control. Data are presented as the mean ± SD. *p < 0.05, **p < 0.01, ***p < 0.001, ****p < 0.0001, NS: not significant. The above experiments were independently performed three times.

In addition to playing an important role in osteogenesis, osteoprogenitors are also a vital source of VEGF. Hu and Olsen found that the osteoblastic lineage (including osteoprogenitors, preosteoblasts, and mature osteoblasts) is an important source of VEGF at the site of injury in cortical bone defects (K. Hu and Olsen, 2017). VEGF is one of the most important angiogenic factors and it is essential for vascular homeostasis (Olsson et al., 2006; Li et al., 2013). In this regard, we investigated VEGF secretion by osteoprogenitors before and after Rg1 treatment. The results of the qRT‒PCR and western blot experiments suggested a common trend (Figures 4A,B). The addition of Rg1 under normal conditions significantly increased intracellular VEGF expression levels. The expression of VEGF was much lower in the high glucose group compared to the control group, but it was raised when Rg1 was given to the high glucose culture. Osteoblastic lineage can guide vascular sprouting through high secretion of VEGF and promote the generation of new blood vessels (Gerber et al., 1999; K. Hu and Olsen, 2016). New blood vessels remove metabolic waste products, increase the supply of oxygen, nutrients, and minerals required for osteogenesis, and may recruit osteoprogenitors to the site of injury, further supporting angiogenesis and osteogenesis through positive feedback loops.

### 5.4 Ginsenoside Rg1 enhanced the proliferation ability of HUVECs exposed to high glucose and promoted their secretion of the osteogenesis-related factors Notch1 and Noggin

**FIGURE 5:**
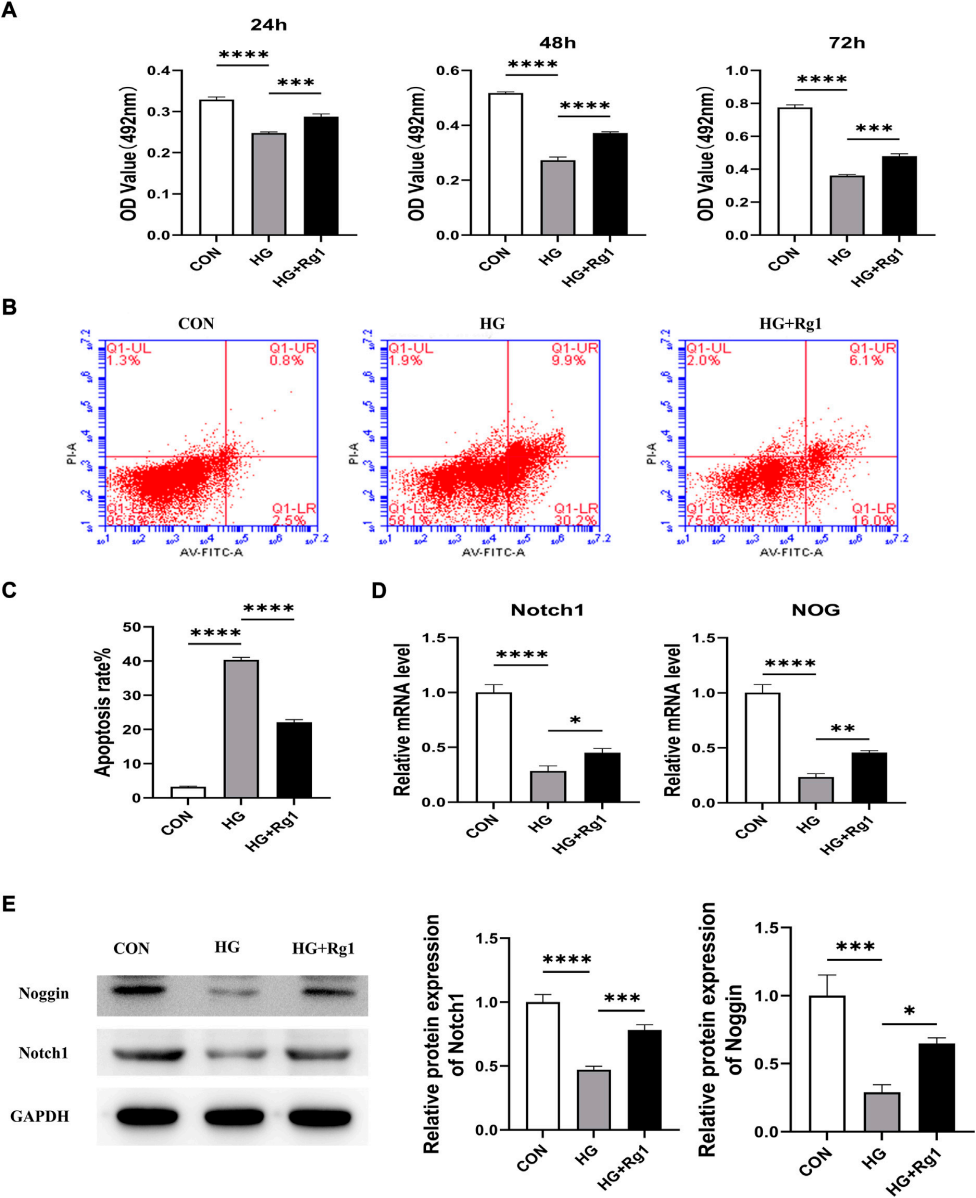
Ginsenoside Rg1 promotes the proliferation of HUVECs and their secretion of Nocth1 and Noggin in high glucose culture. **(A)** MTT assays were used to detect the proliferation of HUVECs by the OD values in each group at 24 h, 48 h, and 72 h. **(B)** Flow cytometry was used to detect the apoptosis of HUVECs in each group at 72 h. **(C)** The apoptosis rate of HUVECs in each group at 72 h **(D)** qRT‒PCR was used to detect the levels of the Nocth1 and NOG mRNA expressed by HUVECs in each group. **(E)** Western blotting was used to detect the levels of the Notch1 and Noggin protein secreted by the HUVECs in each group. The housekeeping gene GAPDH served as an internal control. Data are presented as the mean ± SD. *p < 0.05, **p < 0.01, ***p < 0.001, ****p < 0.0001, NS: not significant. The above experiments were independently performed three times.

Angiogenesis involves endothelial cell (EC) proliferation and, in most developing and regenerating organs, the emergence of endothelial sprouts from preexisting vessels (Wacker and Gerhardt, 2011). To explore the effect of Rg1 on angiogenesis, we examined the effect of Rg1 on the proliferation of HUVECs by MTT assay. According to the experimental findings (Figure 5A), the cell proliferation trend of each group was approximately the same at three time points: 24 h, 48 h, and 72 h. Compared with the control group, high glucose significantly inhibited HUVECs proliferation, but when Rg1 was added, the inhibitory effect of glucose on cell proliferation was alleviated. Rg1 improved cell proliferation inhibition induced by high glucose most significantly at 72 h, so 72 h was used in the subsequent experiments. Flow cytometry further showed that high glucose accelerated apoptosis of HUVECs, while Rg1 significantly alleviated EC apoptosis induced by high glucose (Figures 5B,C).

A large body of evidence suggests that vascular ECs have a significant impact on osteogenic regulation (Ramasamy et al., 2016a). Noggin is a mediator secreted by ECs. It is involved in the regulation of osteoblast function and can promote the proliferation and differentiation of osteoblast progenitor cells as well as the maturation and hypertrophy of chondrocytes. Noggin is positively regulated by Notch signaling (Ramasamy et al., 2014). Notch signaling is a critical component of molecular crosstalk linking angiogenesis, vascular secretory signaling, and osteogenesis (Kusumbe et al., 2014; Ramasamy et al., 2014). It not only plays a decisive role in the differentiation and function of osteoblast lineage cells, but also promotes the proliferation and angiogenesis of EC in mouse long bones (Ramasamy et al., 2014; Ballhause et al., 2021). Notch1 is one of the receptors for Notch. Notch1 signaling positively impacts the fracture healing process (Novak et al., 2020), and inhibition of Notch1 reduces the proliferation and differentiation of osteoblasts (Ann et al., 2011; Zanotti and Canalis, 2014). To further test the effect of Rg1 on ECs, we examined the levels of Notch1 and Noggin in HUVECs. qRT‒PCR (Figure 5D) showed that high glucose inhibited Notch1 and NOG mRNA secretion by HUVECs compared with the control group, and the expression levels of Notch1 and NOG mRNA were significantly increased after the addition of Rg1. The western blot assays for Notch1 and Noggin protein levels expressed in HUVECs showed the same trend (Figure 5E). Thus, we believe that Rg1 can promote the proliferation of vascular ECs exposed to high glucose through activating Notch signaling, which has a positive effect on angiogenesis *in vitro*. Under the stimulation of Notch signaling, Rg1 can further promote ECs to secrete the osteogenic-related factor Noggin, which is conducive to osteogenesis.

### 5.5 Ginsenoside Rg1 can improve bone loss and microstructural deterioration in GK rats

**FIGURE 6:**
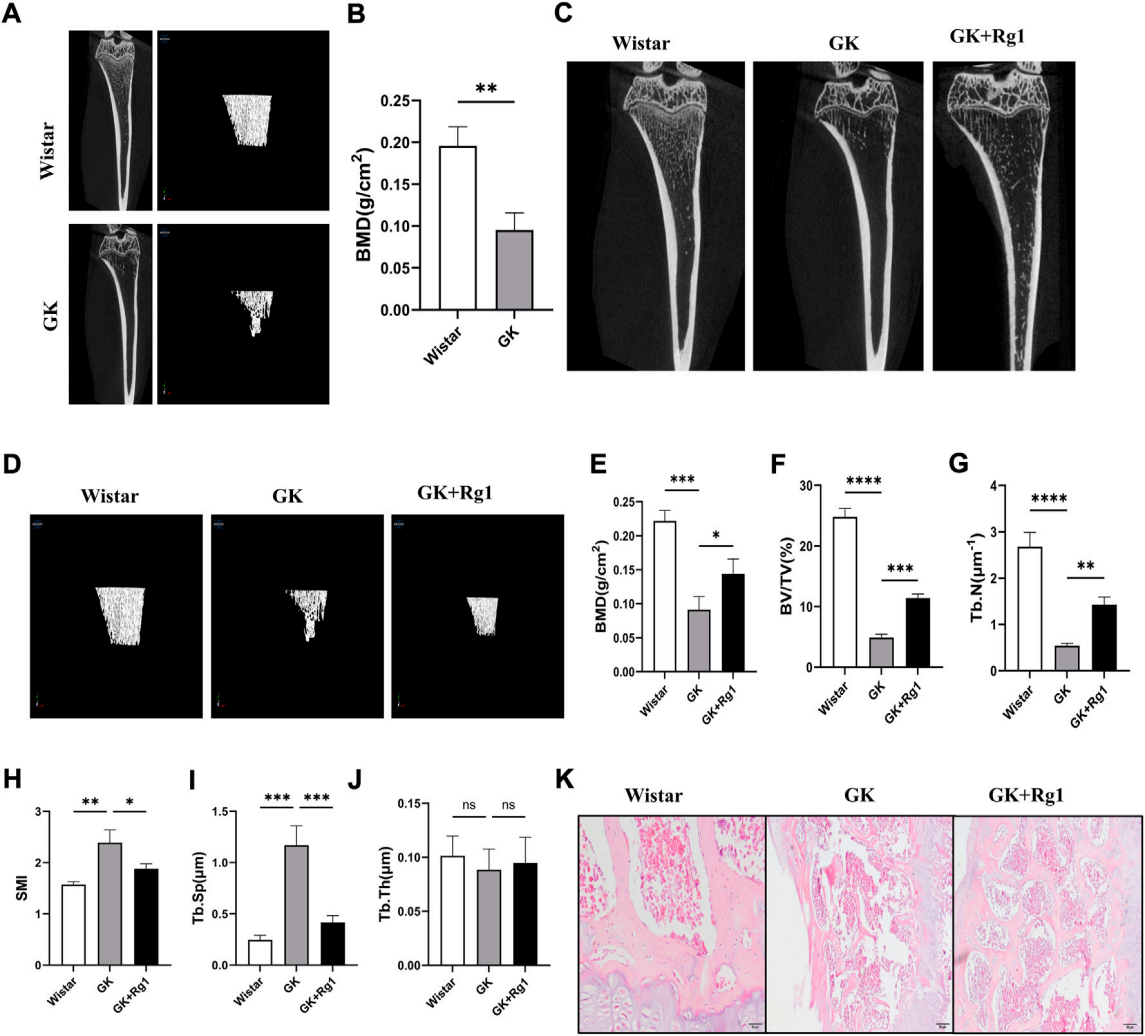
Ginsenoside Rg1 could ameliorate bone mass reduction and microstructural damage in GK rats. **(A)** Micro-CT images of the tibiae of Wistar and GK rats before treatment. **(B)** BMD of the tibia of Wistar and GK rats before treatment. **(C)** Two-dimensional micro-CT images of the tibia of rats in each group after 12 weeks of treatment. **(D)** Three-dimensional micro-CT images of the trabecular bone of rats in each group after 12 weeks of treatment. **(E–J)** Quantitative analysis of BMD **(E)**, BV/TV **(F)**, Tb. N **(G)**, SMI **(H)**, Tb. Sp **(I)**, and Tb. Th **(J)** in the proximal tibia of rats in each group after 12 weeks of treatment. **(K)** H&E staining of the tibial metaphysis in each group. Scale bar: 50 µm. Data are presented as the mean ± SD. *p < 0.05, **p < 0.01, ***p < 0.001, ****p < 0.0001, NS: not significant. *n* = 3. H&E staining experiments were performed independently three times.

To investigate the effect of Rg1 on high glucose-induced bone loss *in vivo*, we used micro-CT to analyze tibiae from GK rats. Micro-CT scans showed that cancellous bone mass was significantly reduced in GK rats compared with Wistar rats (Figures 6A,B), but bone loss was reduced in GK rats treated with Rg1 (Figures 6C,D). Quantitative microstructural analysis also showed that Rg1 treatment for 12 weeks prevented high glucose-induced bone loss, as shown by increased BV/TV, BMD, and Tb. N, and decreased Tb. Sp and SMI in the GK + Rg1 group compared with the GK group (Figures 6E–I). Among them, the Tb. Th index was not significantly different before and after Rg1 treatment (Figure 6J), which may be related to the compensatory increase in trabecular thickness during severe osteoporosis. The H&E staining results further demonstrated the therapeutic effect of Rg1. Compared with Wistar rats, GK model rats had sparse bone tissue, microarchitecture damage, bone thinning, trabecular bone reduction, and an enlarged bone marrow cavity, and after Rg1 treatment, the bone tissue structure became tight, the trabecular bone increased, and the bone marrow cavity decreased (Figure 6K).

### 5.6 Ginsenoside Rg1 promoted the formation of type H vessels and bone formation in GK rats, and increased the expression levels of VEGF, Noggin, and Notch1 in bone tissue

**FIGURE 7:**
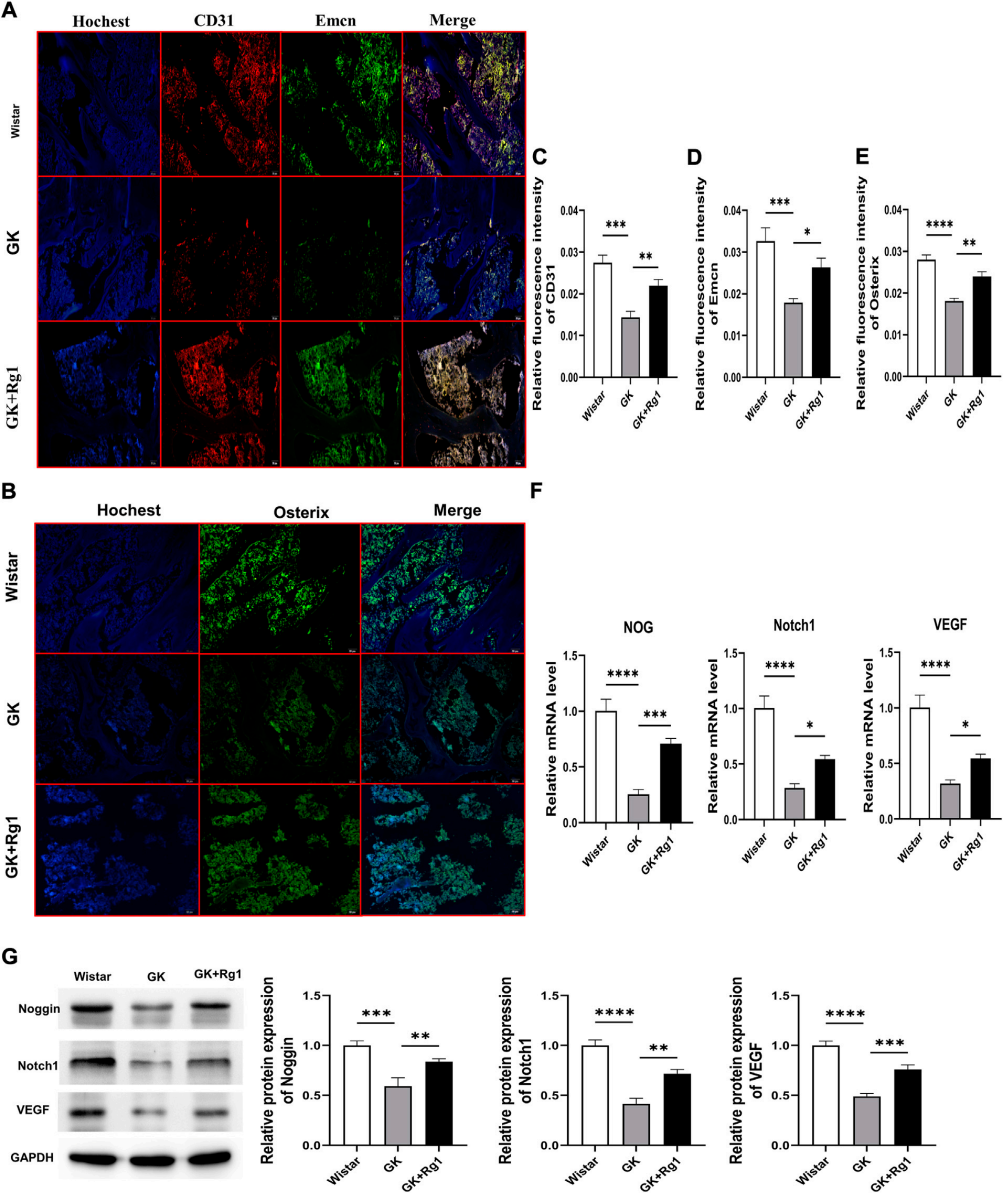
Ginsenoside Rg1 promotes type H angiogenesis and osteogenesis related factor secretion in GK rats. **(A)** Immunofluorescence staining images of CD31 and Emcn in bone tissue of GK rats in each group. **(B)** Osterix immunofluorescence staining images in bone tissue of GK rats in each group. **(C–E)** Quantification of the mean relative levels of CD31 **(C)**, Emcn **(D)**, and Osterix **(E)** in bone tissue of GK rats in each group. **(F)** qRT‒PCR was used to detect the expression levels of the NOG, Notch1, and VEGF mRNA in the bone tissue of GK rats in each group. **(G)** Western blotting was used to detect the protein expression levels of Noggin, Notch1, and VEGF in the bone tissue of GK rats in each group. The housekeeping gene GAPDH served as an internal control. Data are expressed as the mean ± SD. Scale bar: 20 μm *p < 0.05, **p < 0.01, ***p < 0.001, ****p < 0.0001, NS: not significant. *n* = 3. The above experiments were independently performed three times.

We have demonstrated with the above *in vitro* experiments that Rg1 enhances the angiogenic ability of ECs and promotes the upregulation of angiogenesis-related factor (VEGF) expression, indicating that Rg1 may play a potential role in type H angiogenesis, so we verified this conjecture *in vivo* in GK rats. Immunofluorescence staining showed that CD31 and Emcn expression was significantly reduced and that the abundance of type H vessels was impaired in GK rats compared to the Wistar rats (Figures 7A,C,D). At the same time, we also found decreased expression levels of osteoblast-specific transcription factor (Osterix) in the bone tissue of GK rats (Figures 7B,E). However, CD31, Emcn, and Osterix expression levels all increased after 12 weeks of Rg1 treatment (Figures 7A–E). This suggests that Rg1 promotes type H angiogenesis accompanied with bone formation.

Previous studies have shown that both type H angiogenesis and osteogenesis are closely related to VEGF, Notch1, and Noggin (Ramasamy et al., 2014; Fu et al., 2020; Shen et al., 2021). We also found that Rg1 could increase the levels of angiogenesis and osteogenesis by promoting the secretion of the above factors under high glucose exposure *in vitro*. Therefore, we analyzed the expression levels of Notch1, Noggin, and VEGF in bone tissues of GK rats by qRT‒PCR and western blotting. qRT‒PCR analysis showed that Notch1, VEGF, and NOG mRNA expression levels were significantly decreased in the bone tissue of GK rats and significantly increased after Rg1 treatment (Figure 7F). The western blot analysis was consistent with the trends in the qRT‒PCR data (Figure 7G). This suggests that Rg1 plays an active role in osteogenesis by promoting type H angiogenesis through activation of the VEGF/Notch/Noggin pathway under high glucose exposure.

## 6 Discussion

Bone is a connective tissue rich in large blood vessels and capillaries, and the vascular network can act as a structural template for bone as well as regulate bone mass changes (Y. Peng et al., 2020; Tomlinson and Silva, 2013). The vascular system of bone is essential for bone growth, remodeling, and fracture healing. Angiogenesis is a prerequisite for the healing of skeletal conditions such as fractures and defects (Maes, 2013; Ramasamy et al., 2016b). Vascular injury is a major contributor to many organ dysfunctions caused by diabetes, which are referred to as diabetic vascular complications (Beckman and Creager, 2016; Antonopoulos et al., 2021). Previous studies on diabetic bone vessels have focused on the relationship between vascular injury and abnormal hematopoiesis in the bone marrow, and little attention has been given to the potential association between the bone vascular system and bone mass lesions (Fajardo, 2017; Shanbhogue et al., 2017). However, there is increasing evidence that microangiopathy has direct deleterious effects on bone (Oikawa et al., 2010; J. Peng et al., 2016). Type H vessels are specialized capillary subtypes that have been shown to couple angiogenesis and osteogenesis in rodents and humans (L. Wang et al., 2017). They are located in the bone marrow near the growth plate and represent the critical component of the metabolically specialized bone microenvironment with the privilege of obtaining nutrients and oxygen, thereby promoting the growth potential of peripheral perivascular cells (Ellis et al., 2011; Kusumbe et al., 2014; Langen et al., 2017; H. Xie et al., 2014). It has been proposed that human type H vessels are sensitive biomarkers of bone mass and early indicators of bone loss (L. Wang et al., 2017).

It is increasingly recognized that changes in bone mass coincide with the number of type H vessels in aging and ovariectomized (OVX) mice as well as in elderly and postmenopausal osteoporotic individuals (Kusumbe et al., 2014; L. Wang et al., 2017; Xu et al., 2018; Zhao et al., 2012). Recently, it has been shown that osteoblasts may lose the support and regulation of type H vessels when the bone vascular system is affected by metabolic disorders in diabetes, resulting in osteogenic disorders and increased bone fragility (X.-F. Hu et al., 2021). Our results also showed that CD31^−^ and Emcn-labeled type H vessels were significantly sparse in the bone tissue of diabetic rats, and cytological experiments also confirmed that vascular ECs exposed to high glucose had decreased proliferation and increased apoptosis. At the same time, the bone density of diabetic rats decreased significantly, the bone structure was destroyed significantly, and osteogenesis reflected by Osterix labeling decreased. The deterioration of cancellous bone that we observed in GK rats is similar to the findings of other investigators (Y. Liang et al., 2019), suggesting that type H vascular injury during diabetes may be associated with reduced bone mass and osteogenesis. In view of the effect of high glucose on type H vessels and the effect of type H vessels on bone regeneration, we believe that promoting type H angiogenesis may be an ideal strategy to prevent diabetic bone loss.

Among the more than 30 different ginsenosides, ginsenoside Rg1 is one of the most abundant and active components (Radad et al., 2004). Rg1 promotes osteogenic differentiation of a variety of cells. Rg1 treatment upregulated the transcription of osteogenic genes to promote osteogenic differentiation in human dental pulp stem cells and altered gene expression profiles (P. Wang et al., 2014). Wang et al. found that Rg1 activates Nrf2 signaling to regulate osteogenic differentiation of bone marrow mesenchymal stem cells and protects the osteogenic lineage (Hou et al., 2022; P. Wang et al., 2012). Rg1 also inhibited osteoclast differentiation and maturation and reduced destruction of articular cartilage in collagen-induced arthritis mice (Y. Gu et al., 2014). Rg1 treatment has positive effects in both fracture models and ovariectomy-induced osteoporosis rat models (Gong et al., 2006; Y. Gu et al., 2016). However, no studies have been published to date on the protective effect of Rg1 in diabetes-induced osteoporosis.

We investigated the ability of Rg1 to promote type H angiogenesis. Angiogenesis involves the proliferation and migration of ECs, as well as eventual capillary tube formation and the upregulation of some angiogenesis-related factors *in vivo* (Ucuzian et al., 2010). We used HUVECs to determine whether Rg1 could promote vascular EC proliferation. The MTT assay and flow cytometry showed that Rg1 treatment significantly increased EC proliferation and inhibited EC apoptosis induced by high glucose, suggesting that Rg1 can promote angiogenesis. VEGF is one of the major regulators of vascular growth. It has the capacity to control crucial stages of angiogenesis, including the proliferation and migration of ECs (Blázquez et al., 2004). Overexpression of VEGF and its receptors promotes angiogenesis, and inhibition of VEGF blocks angiogenesis (Ferrara, 2004). In this study, we found that high glucose increased the apoptosis of osteoprogenitors, inhibited VEGF secretion from osteoprogenitors, and hindered angiogenesis. However, VEGF gene and VEGF protein levels were significantly increased in osteoprogenitors treated with Rg1 compared with the control group. This is consistent with other investigators reporting that ginsenoside Rg1 promotes VEGF synthesis and thus promotes angiogenic activity (Leung et al., 2006; Chen et al., 2019). We therefore hypothesized that Rg1 has the same effect on type H vessels. *In vivo*, we performed immunofluorescence analysis of CD31 and Emcn. The results showed that CD31 and Emcn expression levels were increased after 12 weeks of Rg1 treatment compared with the GK rat group, verifying the effect of Rg1 on promoting type H angiogenesis.

Notch signaling has been reported to be involved in type H angiogenesis (Kusumbe et al., 2014; Ramasamy et al., 2014). Activation of Notch signaling in bone ECs promotes EC proliferation in the columns of type H vessels and increases the abundance of type H vessels (Ramasamy et al., 2014). Notch signaling also controls the release of the vascular secretory factor Noggin. On the one hand, Noggin stimulates the differentiation of osteoprogenitors, affects trabecular bone mass, and growth plate morphology. On the other hand, Noggin promotes the maturation and hypertrophy of chondrocytes, thereby affecting angiogenesis through VEGF-A expression in hypertrophic chondrocytes (Ramasamy et al., 2014). VEGF is involved in bone formation in addition to angiogenesis (Yang et al., 2012). Loss of VEGF-A expression in osteoprogenitors inhibited their differentiation into mature osteoblasts, resulting in reduced bone density (Liu et al., 2012). Previous studies have also shown that VEGF, in addition to increasing the activity of ECs and promoting angiogenesis, directly enhances the recruitment and differentiation of osteogenic progenitor cells, thereby regulating the morphogenesis of the growth plate and promoting fracture healing (Mayr-Wohlfart et al., 2002; Keramaris et al., 2008). In conclusion, the VEGF/Notch/Noggin pathway emphasizes the coupling between angiogenesis and osteogenesis, reflecting communication between ECs and osteocytes. This in-depth understanding of the role of angiogenesis-osteogenic coupling in the bone microenvironment may aid in the development of new diagnostic biomarkers and therapies to treat bone lesions, such as osteoporosis or osteonecrosis.

EC-specific inactivation of Notch signaling through inactivation of Dll4 or Rbpj, which encodes RBP-Jκ, an essential mediator of Notch-induced gene transcription, leads to a reduction in angiogenesis, loss of type H vasculature, and reduced bone formation (Ramasamy et al., 2014). Deficiency of Notch activity also significantly decreases Noggin expression by vascular endothelium, resulting in impaired osteogenic and chondrocyte differentiation function in mice (Ramasamy et al., 2014). Another study showed that the reduction of type H vessels and the reduction of serum VEGF and Noggin levels in hindlimb-unloading (HU) mice were parallel to bone loss (S. Liang et al., 2021). We also found that high glucose inhibited the expression of Notch1, Noggin and VEGF in GK rats, which was more intuitively reflected by the decreased expression levels of CD31, Emcn and Osterix in the bone tissue of GK rats. These results suggest that high glucose may affect angiogenic osteogenic coupling by inhibiting the VEGF/Notch/Noggin pathway and ultimately accelerate osteoporosis. All the above data indicate that Rg1 plays a role through VEGF/Notch/Noggin pathway. However, other factors that may be involved in the preventive effect of rg1 on high glucose-induced bone loss, except VEGF, Notch1, and Noggin, remain to be elucidated. We will conduct more in-depth studies of Rg1 in the future including experiments in toxicology.

In conclusion, the findings of this study suggest that Rg1 plays an active role in diabetic osteoporosis by positively regulating the Notch pathway, stimulating VEGF and Noggin signaling, reversing the damage inflicted by high-glucose exposure on osteoprogenitors and ECs, promoting the secretion of factors related to angiogenesis and bone formation, increasing the number of type H blood vessels, and enhancing the coupling between angiogenesis and osteogenesis. To our knowledge, this is the first study to report that Rg1 can interfere with the progression of diabetic osteoporosis by promoting type H angiogenesis and modulating vasculogenic and osteogenic coupling, expanding the therapeutic range of Rg1. Finally, these findings from cytology and animal models need to be validated in human samples, and clinical analysis of human bone specimens should be performed in the future.

## Data Availability

The original contributions presented in the study are included in the article/Supplementary Material; further inquiries can be directed to the corresponding author.
