# Supplementary material for: Ginsenoside Rg1 interferes with the progression of diabetic osteoporosis by promoting type H angiogenesis modulating vasculogenic and osteogenic coupling
Source: Front Pharmacol. 2022 Nov 17;13:1010937. doi: 10.3389/fphar.2022.1010937 (PMC9712449; doi:10.3389/fphar.2022.1010937)
Supplement: Supplementary file 1 [file Table1.docx]

Supplementary Material

**Supplementary Table S1.**

**TABLE S1** | Exploration on the Effective Concentration of Ginsenoside Rg1.

| **Drug concentration（μM）** | | **OD492nm** | | | **Inhibition rate%** | **SD** |
| --- | --- | --- | --- | --- | --- | --- |
| Ginsenoside Rg1 | 0μM | 0.451 | 0.443 | 0.429 | 0.00 | 0.00 |
|  | 5.15μM | 0.438 | 0.472 | 0.428 | -1.53 | 6.46 |
|  | 10.3μM | 0.461 | 0.429 | 410.475 | -4.38 | 9.40 |
|  | 20.6μM | 0.467 | 0.468 | 0.459 | -7.20 | 2.37 |
|  | 41.2μM | 0.497 | 0.439 | 0.484 | -9.84 | 9.75 |
|  | 82.4μM | 0.482 | 0.490 | 0.521 | -17.35 | 10.26 |
|  | 164.8μM | 0.531 | 0.470 | 0.507 | -18.67 | 9.16 |

**Supplementary Figure S1.**


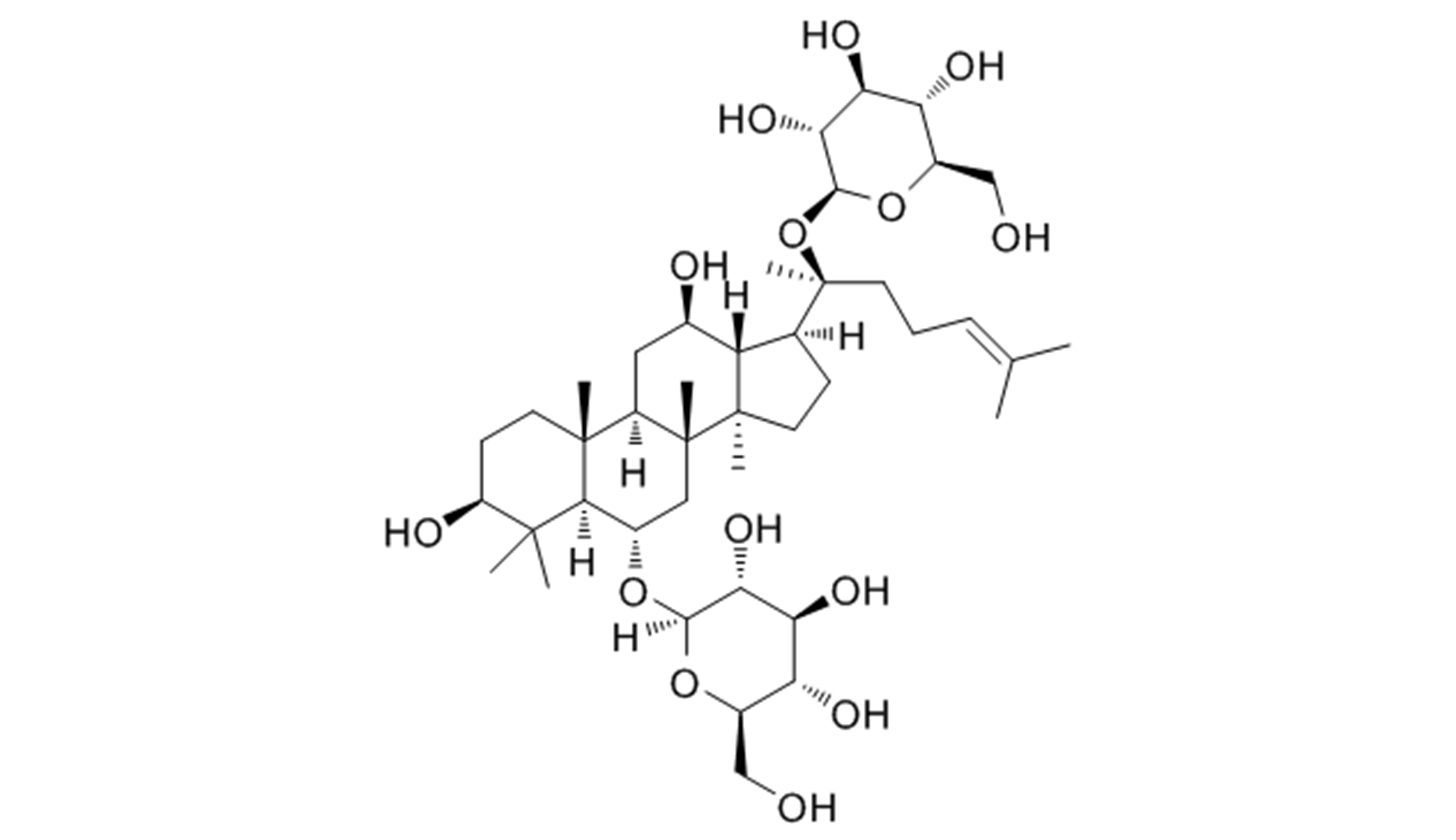


**FIGURE S1 |** Molecular Structure Formula of Ginsenoside Rg1.
